# Imagining the city in lockdown: Place in the COVID-19 self-recordings of the Lothian Diary Project

**DOI:** 10.3389/frai.2022.945643

**Published:** 2022-12-05

**Authors:** Claire Cowie, Lauren Hall-Lew, Zuzana Elliott, Anita Klingler, Nina Markl, Stephen Joseph McNulty

**Affiliations:** Linguistics and English Language, School of Philosophy, Psychology and Language Sciences, The University of Edinburgh, Edinburgh, United Kingdom

**Keywords:** COVID-19, pandemic, lockdown, narrative, chronotope, diary, time, place

## Abstract

The COVID-19 pandemic brought about a profound change to the organization of space and time in our daily lives. In this paper we analyze the self-recorded audio/video diaries made by residents of Edinburgh and the Lothian counties during the first national lockdown. We identify three ways in which diarists describe a shift in place-time, or “chronotope”, in lockdown. We argue that the act of making a diary for an audience of the future prompts diarists to contrast different chronotopes, and each of these orientations illuminates the differential impact of the COVID-19 lockdowns across the community.

## Introduction

The COVID-19 pandemic brought about a profound change to the organization of space and time in our daily lives. The onset of the pandemic saw restrictions to mobility at both local and global levels, severely impacting everything from daily commutes to international travel. In the lockdowns, we spent the longest periods of our lives in the smallest amount of space. Our interactions with the physical environment changed. For many, lockdowns entailed shifts to learning and working from home, but the perception of space also changed for key workers working outwith the home. The subjective experience of time began to change as well, apparently shrinking and expanding. It is not surprising that many people began to rethink their understanding of place, by which we mean space imbued with meaning and emotional attachment (Cresswell, 2015).

The Lothian Diary Project collected audio and video self-recordings about COVID-19 from May 2020 to July 2021. The only criterion for participation was residency in Edinburgh or the Lothian counties (Scotland), so contributors may have been primed to reflect on place, in particular. The present paper describes how lockdown affected diarists' experience of place and time, and how they represent this change. To capture the distinct discourses of place that appear in the narratives, we draw on Bakhtin's notion of the chronotope (1981). Chronotopes are “descriptions of the looks, behaviors, actions and speech of certain characters, enacted in specific timespace frames” (Blommaert and De Fina, 2017, p. 3). We argue that diarists use chronotopic shifts to express emotion and to signal their attitude to social restrictions. Our analysis of a specific subsection of participants finds three distinct chronotopic shifts expressing how the experience of space and time changed for these diarists during lockdown. International students, in particular, were found to have shifted from experiencing the city solely as a place to study toward claiming ownership over it as a place to live. Secondly, retirees notably shifted their busy schedules of activities from the physical world to the virtual world, finding it a satisfactory and, for safety reasons, necessary substitute. And finally, men resident near the city center expressed a sense of having lost freedom of movement and choice as a result of lockdown.

## The Lothian Diary Project

A full description of the Lothian Diary Project corpus and the methods of its collection is available in Hall-Lew et al. (2022). Participation was open to any resident of any background (and any language) residing in the city of Edinburgh, Scotland's capital (2020 population, 488,050) and its surrounding counties, known as the Lothians (2020 population, 413,405). The LDP is comparable to other COVID-19 diary projects that have a geographical locus (e.g., Sneller et al., 2022) and distinct from diary projects that have a wide geographical distribution (e.g., Faircloth et al., 2022). The data collection process was geared toward public engagement: prioritizing financial support for participants and local charities, offering free citizen science training and awards for young people, producing a report and roundtable event for the Scottish Parliament, and creating an oral history archive. The multidisciplinary academic team of sociolinguists, data scientists, political analysts, and health scientists are using the resulting corpus (Hall-Lew et al., 2021) to address a range of theoretical and methodological questions (Markl and Lai, 2021; Hall-Lew et al., 2022; Markl, 2022). For the purposes of the chronotopic analysis we employ in this paper, it is important to note that all of the diaries analyzed in the current paper were recorded between May and July 2020 (the latter months of the first lockdown in the Lothians), unlike others in the full sample which were made between August 2020 and July 2021, a period during which public policies and public attitudes toward COVID-19 shifted considerably.

Recruitment of the diarists took place through word of mouth, radio and social media advertisements, and in some cases through charity partners who were working with those most severely affected by the pandemic (e.g., unhoused people, people usually reliant on various support groups and social services which were disrupted by the pandemic). These local charities recruited their clients for participation and assisted with recordings in exchange for extra financial compensation. Participants recorded themselves speaking in response to questions posted on the project's website:

### Audio/video diary prompts

- How has your life changed during lockdown?
- What was a typical day like before lockdown, and what's it like now?
- What's been the hardest part for you during lockdown?
- Have you learned any new skills or taken up any new hobbies?
- Have you been working from home? Has it been challenging?
- Have there been any bright spots about the lockdown?
- Who are you in lockdown with, and how are they doing?

Some answered these prompts directly, like a self-interview; others addressed them more abstractly. On submission, participants could opt in to the inclusion of their diary in an oral history archive. The diaries were transcribed automatically and then checked by hand. Ongoing analysis of audience design (Hall-Lew et al., in prep) finds a wide range of genres represented by the collection of what we call “diaries” for simplicity's sake. These include video recordings stylized like vlogs (Pihlaja, 2018), recordings framed as broadcasts (e.g., “shout out to the NHS”), and recordings designed for a historical archive (e.g., “maybe 1 day when you hear this you'll get an idea of what it was like in 2020”). However, most entries were less overtly oriented to any particular audience and therefore more “diary”-like in style, even though they were one-off accounts instead of a more conventional personal diary with regular entries. For example, roughly half of the contributions begin with a greeting (“Hi” etc.), and for most of them this is the only audience-directed utterance in the recording; for the other half, there are none at all. After recording the diary, participants uploaded the file and completed a survey of demographic questions and questions about lockdown experience.

For this paper, we focus on the transcripts of those diarists who were living alone at the time of making their diary (*N* = 36 of 195). The effects of isolation were a common topic in the early days of the pandemic and it will take many years before we fully understand them (see Ganesan, 2021). We decided to start our analysis with the “living-alone” sample, as their diaries are more reflective of their personal experience—potentially including senses of place and conceptions of time-space—rather than focused on the changing interpersonal dynamics of members of a household. At the outset of our more in-depth analysis of the diaries, we wished to avoid singling out a demographic. The “living alone” sample is mixed in terms of age, gender, sexuality, ethnicity, and residential status.

We are able to draw the sample reliably because diarists had to provide information about occupancy on the accompanying survey. However, most of the diarists in the sample announced their lone status very explicitly. Even though the question about cohabitants is the last of the prompts, diarists tend to announce it at the start of their diary, as part of a surprisingly uniform introduction routine. A typical diarist in our subsample will give their name, sometimes their area, note that they are living alone, and express themselves fortunate to not be as adversely affected by illness as others, before describing their lockdown experience. In the section Place in the LDP and in the “living alone” diaries we elaborate on the role of place in the diaries, and especially in the subsample used for this paper. In the section Chronotopic analysis we outline some of the applications of “chronotope”, especially in narrative analysis. In the section Shifts in chronotopic orientation, as a unit of analysis, the chronotope allows us to explore different identities that are linked to representations of place. We will see that the act of making a diary (possibly for an audience of the future) prompts diarists to produce different chronotopes, and in our analysis we attempt to map the relations between those chronotopes. In the Discussion section, we take into account the material conditions of the diarists, and their demographic characteristics, to understand the ideological positioning that is signaled by chronotopic shift.

## Place in the LDP and in the “living alone” diaries

In our early exploratory analysis of the LDP data with the the Edinburgh Geoparser^1^, a natural language processing tool developed to identify place name references in English text^2^, we found that the majority of place name mentions (53%, *n* = 438) were actually of locations outside of the UK, as contributors talked about video calls with friends and family abroad, and canceled travel plans. Certainly the LDP diaries, like any discourse of the pandemic, reflect the global increase in virtual connectivity accompanying the global loss of physical mobility (Scott et al., 2022).

Given that residency in Edinburgh and Lothians was a requirement of participation, there is perhaps less mention of places in Edinburgh and the Lothians than we might expect: 31 mentions of locations within Edinburgh, 122 mentions of Edinburgh itself, and 18 mentions of locations in the Lothian area. As Cresswell notes, place is typically described in terms of “[n]eighbourhoods, villages, towns and cities”, because they are “small in scale, but not too small” (Cresswell, 2015, p. 18). Neighborhoods do not appear to dominate these discourses of the pandemic, in contrast to their prominence in the interviews of previous sociolinguistic projects in Edinburgh (e.g., Esling, 1978). Given media reports of real and perceived increases in community cooperation during the first lockdown^3^, we expected to find stories of specific neighborhoods pulling together. Such accounts are not prevalent, however. We found only one in the living-alone sample (1), and the location is not in fact mentioned.

(1) Jess^4^
“I worried about my wee town that there wasnae any community spirit and, you know, we just didnae help each other oot and scratch each other's back and do wee favors. You know like, if you're going doon to the shop you w- and get Wee Maggie a loaf of bread and, a pint of milk and, you know, I was worried that all of that was disappearing and our world was fully technology and we were becoming quite cold and that and not acting properly with people. But through this—it it shows me that our wee community can rally together and become really, really strong.”

With three exceptions, the diarists of the “living alone” sample started with a mention of their inner city area or suburb, and a mention of Edinburgh, e.g., “I live in Leith, in Edinburgh”. University students referred to the “University of Edinburgh” or simply “the university”. This suggests that they are primed to discuss the city, although we will see that the *ways* in which they talk about the city vary considerably.

Most of the living alone sample recorded diaries from their homes. Two outliers were Jonathan and Veronica who were in temporary accommodation at the time of recording. Both experienced very extreme and literal displacement during lockdown. For Jonathan, who suffered a psychological breakdown during lockdown and became homeless, there is a stark contrast between Edinburgh and “wandering the country” (2).

(2) Jonathan
“During the lockdown I was actually homeless and I spent quite a majority of the first part of it wandering the country. I was suffering a breakdown and I lost all my possessions. Eventually when I came to Edinburgh I was hospitalized, suffering from extreme breakdown and diagnosed with bipolar. Since then I was passed on to the council and they're providing me with temporary accommodation currently.”

For Veronica, a recovering addict who relied on charities for food and temporary accommodation, lockdown meant daily travel right across the city, first on foot, and then by bicycle (3).

(3) Veronica
“The hardest part of lockdown for me […] physically to walk three and a half miles to get food and three and a half miles back. I struggled but I did it and I never thought I would have been able to do, especially going up the hills and everything in Edinburgh. But I did it, slowly but surely, I did it. […] Again, going back to the charities who helped me with that donated bicycle, the bicycle was the real gift. I took to it really well. Again, a major struggle going up the hills, but I just persevered and had a lot of fun coming back down them and my food would still be hot by the time I would get home.”

Veronica's experience of the city is evidently transformed. However, the shifts in time-space that the remainder of the “living alone” diarists describe, emerge from a highly confined and isolated experience, often driven by the shift of work, and other routines, into the home and online^5^ We will see that diarists express these changes in space and time in different ways, and with different stances. Their representations of the city are abstract, often to do with desire, rather than events or stories. This quality means that chronotopic analysis is most suitable for our analysis of place. In the section Chronotopic analysis, we review studies that have explored the role of chronotopes (and related concepts such as figures of personhood) in narrative.

## Chronotopic analysis

Bakhtin (1981, p. 84–85) use of *chronotope* or “timespace” is concerned with “the intrinsic connectedness of temporal and spatial relationships,” initially focused on literary analysis. This concept is connected to Bakhtin's theory of *heteroglossia*, the multivocality (i.e., complex indexicality) of every act of speaking. Every utterance occurs in a timespace that constrains or enables its legibility. A literary analyst can use chronotopes to identify when different fictional events are in “dialogue” with one another. Extending Bakhtin's application in literature (Blommaert and De Fina, 2017, p. 3), the concept was taken up in sociology and anthropology with a focus on social types (e.g., Goffman, 1981), or as a “nexus [...] of time, space, and identity” (Schiffrin, 2009, p. 421). The imagined speaker (narrator or narrated) of a timespace frame has been described as a “figure of personhood” (Agha, 2007). This figure of personhood (and thus the chronotope), which is not always overtly referenced, can be indexed through appearance, behavior, demeanor, character and practice (Park, 2021, p. 49–51). Something as small as a certain phrase (Blommaert and De Fina, 2017) or the pronunciation of a name (Rosa, 2016) can index a chronotope.

Here we are most interested in approaches that foreground a geographic location or place in the chronotope. Britt (2018) examines “discourses of place” and chronotopic representations of Flint, Michigan, “that cast the locale (Flint) as a certain type of place (i.e., “apocalyptic” and in decline) populated by a certain type of person … at a certain moment in time” (Britt, 2018, p. 253). This chronotope is the way residents of Flint depict the views of outsiders, and they then counter this view in their own narratives, distancing the views of the outsider through the use of reported speech.

Narrators use chronotopes to relate everyday human experience to what (Bakhtin, 1981, p. 208) called “the collective historical life of the social whole” (Pritzker and Perrino, 2021, p. 367). LDP contributors reflect on the everyday reality of the lockdown, but their act of contributing a diary to the project orients them to the collective historical life of the social whole not just of Edinburgh, but also the pandemic. Because these are snapshots, taken toward the end of the first lockdown, they differ from the “coronatopes” universally experienced over the course of the pandemic, such as the daily graph of cases and deaths, the calculations of quarantine periods, the passage of subsequent lockdowns, even the order of the variants (Weichselbraun, 2022).

One might expect the LDP diaries to be comparable to other chronotopes of crisis, for example showing a chaotic narrative structure (Goldstein, 2012). However, in a separate analysis (Hall-Lew et al., in prep) we find very few chaotic narratives among the LDP contributions. We suspect that this is because the nature of the crisis in most cases is more existential than imminent: everything is happening and yet nothing is happening, because at the time of speaking the speakers' movements are (unusually) restricted. None of the LDP contributors, and none of their immediate family, were suffering from COVID-19 at the time of recording. The chronotope is specifically about the conditions of lockdown, not crisis.

This is contrast to the COVID-19 diaries collected by the Mass Observation Archive which show an “ebb and flow of consciousness” (Pattrick and Scantlebury, 2021), as they were collected collected at points over a period (Barnett and Clarke, 2021; Sneller et al., 2022), when experiences with COVID-19 were constantly in flux.

Among the contributors to the Lothian Diary Project during the first lockdown we see connections to other chronotopic representations of cities as being at a “standstill”. As Weichselbraun (2022) observes, “stopping movement in space also somehow produced a sense of stopping time, by stopping/interrupting our quotidian activities, the streets were dead”. The main chronotopic shift into lockdown was that time seemed to change quality, or operate differently, than it had before. Weichselbraun notes that her students in Vienna divided time into before and after lockdown. The LDP website prompted diarists to distinguish between pre-lockdown and lockdown, motivating the production of opposing chronotopes, but also motivated by our research team's own experiences. “Before lockdown” and “lockdown” become distinct chronotopes in which space and time are organized differently, much in the way that previous work has shown between different cultures (Schiffrin, 2009, p. 423) or different historical genres (Bakhtin, 1981; Park, 2021, p. 53).

In all the recordings analyzed here, speakers employ shifts in tense and shifts in deixis to “zoom in” and “pan out” of time and space (Pritzker and Perrino, 2021). In doing this they move from their present reality closer to and further away from imagined ideas of Edinburgh and their local area. De Fina (2021) describes how “through narratives, participants bring to bear in their present interactions worlds and historical moments that belong to different geographical and temporal scales” and in so doing “create new understandings of reality and also new patterns of social interaction” (2021, p. 60). Pritzker and Perrino (2021) show how the narrator Moreno, an Italian fashion executive, shifts between chronotopes, interweaving his company's history and his family's history with an “imagined collective identity” (p. 371). Moreno uses biological metaphors such as “it's in our DNA” to connect his personal body to the public world of Mantua and of the “Made in Italy” national brand (Pritzker and Perrino, 2021, p. 368–375). We notice a similar process in which LDP diarists take affective stances (Du Bois, 2007) toward pre- and post-lockdown chronotopes, constructing an ideological position on the pandemic. Park (2021, p. 48, 50) notes how understanding imagined figures of personhood and their chronotopes, with reference to the material conditions of the speaker, facilitates a critical analysis of the political processes underlying society. As Creswell says: “Place, at a basic level, is space invested with meaning in the context of power” (2015, p. 19).

Our observations of the time-space frames by diarists in the LDP show that they typically produce at least two chronotopes in their narrative, imaginings of the city prior to lockdown and imaginings of the city in lockdown. We are interested in this distinction, or the shift between these chronotopes, and what ideological positions it makes possible for diarists: in particular, whether they are supportive of the government's lockdown policy, or not. In the following analysis of the 36 diarists living alone, we have identified three of these shifts in chronotopic orientation, which are not exhaustive, but seem to represent three quite different kinds of experiences of lockdown.

## Shifts in chronotopic orientation

### Edinburgh as a place to study vs. Edinburgh as a place to live

In the living-alone sample, nine diarists had a temporary residential status (as indicated on the accompanying survey), and all but one of them talked about a fresh encounter with their material environment, in which they renegotiated their position toward the city. Tengfei, a doctoral student, says “the good thing is Edinburgh became very quiet and I can just walk around the city. Uh, you know, enjoy the city and see”. Shuxin “felt grateful that I'm living in a city, Edinburgh, where it's not so chaotic or so huge as in London”. A more elaborate description of the new cityscape by Catherine, a student from Brazil, involves many shifts in perspective (4). The sea is brought closer (“just half an hour away”) but then walks open up with parks, and parks open up with lakes. We are then brought back through proximal deixis to “this very tiny room”.

(4) Catherine
“I think that the lockdown, er, helped me to truly know the city I have been living for almost a year now. When I arrived in Edinburgh last September to pursue a master's degree, I didn't have many chances to visit spots other than the main touristic attractions, and during my academic year my usual route was from the student accommodation to George Square, stopping by the supermarket, and I also used to go to the swimming pool three times a week. It felt like I was an international student living in, in a city, with a well-known university. But during lockdown, I committed myself to go for a walk, every day, in order to exercise, and then I had a chance to know a city that was totally new for me. I discovered that my place was just half an hour away from the sea. I found lovely {parks} in Water of L- Leith walks, lakes that I've never seen before in Holyrood Park, and for the first time since I arrived, I really felt part of this place. … I think that lockdown was certainly a challenge for me because I was away from home, and I had to spent most of my time in this very tiny room. But, if it wasn't for it, I wouldn't have a chance to really experience Edinburgh, and feel like this place, is also mine.”

Another student, Patricia, a postgraduate student from Hong Kong originally; living in the Lothians since 2017, speaks of a new attention to her environment, and in the same way, reflects on how this makes her feel about Edinburgh (5). She talks about a feeling of “how it is to live in Edinburgh”, creating a contrast between studying in the city and living in the city.

(5) Patricia (translated from Cantonese)
就其，某程度上多、多去，真去感受Edinburgh生活感
It turned out that, to a certain extent, I had more time and opportunity to feel how it is to live in Edinburgh
即比留意多，即呢境、身人、身啊,
I paid more attention to the environment, the people around and the shops around.
即就算人，都多去察,
Even when the shops were closed, I would still observe that
哦，原呢度有，哦到lockdown完都想、想去呢睇一睇
oh, there was such a shop here, I would want to come here and have a look after the lockdown.
我得呢可以lockdown, 其中一改我地方,
I would say this is a change that I had during the lockdown,
就係,就係我會依家好享受即係自己一個落街行
I really enjoy walking alone on a street now,
就好似好漫目的行
walking as though there isn't a purpose,
粹for想更加感受下呢城市
Purely for experiencing/feeling this city more.

For newcomers/temporary residents, because their primary locus is elsewhere, they may previously have been inhibited from exploring in this physical, sensory way. Catherine, too, draws a parallel between two city-chronotopes (see Pritzker and Perrino, 2021, p. 380 on parallelistic structures). One is the city with a well-known university, and the figure of the international student, where time and space is chunked into student-related activities. These take place in, for example, the library, and “touristic attractions”, where the emphasis is on socializing rather than sensory experience. Timespace previously distanced from the material world because of this international student lifestyle and identity, shifts to become more concrete, or closer to the timespace of the material world (Park, 2021, p. 53).

In the chronotope of lockdown conveyed by Tengfei, Shuxin, Catherine, and Patricia, the figure of the international student is backgrounded, and the figure of an Edinburgh resident emerges. In contrast are international students who do describe the lockdown chronotope in terms of loss rather than gain. Rajesh, a postgraduate student from India, describes a vacuum created by the lockdown:

(6) Rajesh
“This is too much to handle. I can't go to the library, and I avoid gatherings and everything, can't do much of things. There's no lectures, there's no activities, no football, no sports, nothing.”

Rajesh is oriented to a very different lockdown chronotope than the other international students see so far. His description in (6) frames the lockdown period as deeply overwhelming, “too much to handle.” And yet that which is “too much” is not an overwhelming abundance of something, but the absence of everything. Rajesh's lockdown storyworld is constructed as “nothing”, a nothingness that is discursively enhanced by the parallelism of the four “no X” constructions immediately preceding it. Virtual space is not entertained as a possible new space, but rather the absence of space: “no lectures” erases the existence of online lectures, and “no activities” erases all student-oriented activities that were moved to virtual spaces. Another student, Siu Ming (speaking in Cantonese), says that his friends and classmates “were not used to being alone, staying home all the time, working at home, studying at home etc. I did try my best to help them”. The parallelism of “at home” is similar to Rajesh's parallelism in the way it conveys stasis, but it is less bleak, in that there is a something, and not just nothing. The chronotopic contrast that both Rajesh and Siu Ming orient to frames the lockdown timespace as a loss, whereas the other students frame the lockdown time-space as a gain.

Although Catherine is still an international student at the time of speaking, she distances herself from her life as an international student through use of the past tense (“I was an international student living in, in a city, with a well-known university”). Her de-identification with this figure and its chronotope evidences a shift and re-negotiation of identity (Blommaert, 2015; Blommaert and De Fina, 2017). For both Patricia and Catherine, there is a transition from actions (“my usual route was…”) to experiences (“I discovered…”; “I paid more attention…”) and feelings (“I really felt…”; “I really enjoy…”) and, for Catherine, a transition to a new identity (“part of this place”). When Patricia says she enjoys walking alone, she shifts into the present tense. For these speakers, the lockdown chronotope is characterized by possibility and change. This negotiation is different for Rajesh, who does not identify any new identities available in the lockdown chronotope (“nothing”).

### Physical place can be replaced by virtual space

For many diarists, activities that constitute important parts of their identity have moved online, and this is foregrounded in their diaries. While some students framed this change in a wholly negative way (6), diarists in retirement described the impact of this particular shift quite differently. Previously, few if any of their daily activities and social actions took place in virtual space.

Alistair is an older male who retired to a small town in the Lothians just before the pandemic. Although (like the students above) he is not long in the area, he sees himself as someone who will live out his life there and be an active member of the community. He announces this intention as he opens the diary, and then lists the range and extent of his activities pre-lockdown (7), emphasizing the importance of having a full schedule and “keeping busy”. At the center of this is attending services in the local church. Strikingly, and in contrast to Rajesh's sentiment in (6), Alistair expresses satisfaction that he can continue with the new community that he has acquired and its local character, online, in lockdown:

(7) Alistair
I retired from business recently as now I'm 73 years old and I wanted to contribute to my local community, meet new people as I'm an– as I am a new boy in Haddington and have an interest in activity. I helped at a day care center, and packed food parcels at a nearby food bank in Tranent, and also volunteered as a greeter at the local NHS hospital in Haddington. These activities kept me busy 5 days per week. In addition, other activities centered around the local Holy Trinity church of which I am a new member of the congregation. When the virus struck and lockdown and isolation became the rule I was forced by my age to stop all the voluntary activities. And then the church was ordered to close… The Rector at Holy Trinity started offering Eucharist services on zoom. That was an excellent substitute for attending the service in the church and a fine opportunity to see new friends in the congregation and chat informally after the service time.”

Sheila is also a retiree, and she presents herself in a similar way as someone with a busy life. Although this is not so explicitly linked to place as the speaker from Haddington, she opens by mentioning her suburb of Edinburgh, and certain activities that are attached to very specific locations such as volunteering at “Oxfam bookshop”.

(8) Sheila
“Before lockdown, I led a really busy life. Retirement opened up time for all sorts of groups and classes. I have done history {work}, pottery and gallery tours among others. I am a Taoist Tai Chi instructor and attend classes four times a week as well as traveling in GB and Europe to workshops … Swimming has always been one of my activities. I usually swim three or four times a week… I volunteer in Oxfam bookshop one afternoon a week. Theaters, films, and meals out with family and friends also keep my diary pretty filled. Like many retired people, I wonder where I find time to work … I go to virtual theater, ballet, and opera a couple of times a week. Sometimes I coordinate this with friends and we virtually share a wee glass of wine, or two, if we're watching something on YouTube.”

As Sheila lists her pre-lockdown activities (8) she switches from simple past (“opened up”), to present perfect (“have done”) to the habitual present (“usually swim”). Notably, her lockdown activities are delivered in this same habitual present (“we virtually share”). Alistair uses the simple past for pre-lockdown and lockdown activities. Neither therefore use tense to distinguish between their daily routine pre-lockdown and post-lockdown.

Although in her diary Sheila also talks about missing physical contact, and the difficulties of social distancing with her grandchildren, some aspects of her busy schedule can be moved online. For certain activities such as theater, which are not only for entertainment but for socializing, she creates a parallel structure (Pritzker and Perrino, 2021, p. 380) in her narrative of pre-lockdown and lockdown. Both speakers work to keep their previous schedules the same in lockdown. Space has changed from the physical to the virtual, but the experience of time is presented as staying the same, and the experience of place is presented as being a satisfactory substitute.

### Edinburgh was all about freedom and choice

Andrew, also retired, mentions three pubs in his area by name, following an introduction in which he mentions his area of the city and how long he has lived there (25 years). As a response to the prompt “what have you missed?” he lists places he would visit, even the days of the week that he would visit them, and the activity involved (9).

(9) Andrew
“Erm I miss good beer and, and going to pubs like, er, Sandy Bell's on a Friday night or, er, on Thursday night the Antiquary, and, er, I miss the music that was played there, and I miss reading a newspaper late at night at The Stockbridge Tap, that was another, erm, er, enjoyable thing to do. Trips to the cinema, erm, again this is something which I did quite frequently, maybe at least once a month.”

This is similar to the older speakers in the section Physical place can be replaced by virtual space, with their full and busy lives, but this speaker dwells on activities which can't be replaced with the virtual version, partly because the specific location is constitutive of the activity, and also likely because the communities linked to that are not comprised of known individuals, but shifting populations of similar characters. The timing of these activities is necessarily unstructured so as to provide choice. It is more than just a question of different activity types, however; all of the diarists have a mix of activities. It is not a resistance to technology (Andrew later discusses communicating with friends and family online) or a resistance to socializing (he is in a “bubble”^6^ with a friend). Rather, diarists like Andrew are drawing attention to those activities which cannot be made virtual; aspects of a particular neighborhood chronotope that were lost in lockdown.

The pre-lockdown lifestyles of choice described in this section are of course more characteristic of those living-alone, but urbanization and choice takes on greater significance in lockdown. The COVID-19 lockdown created a chronotopic shift from a place of choice to a place of restriction, and this is seen most acutely for those living closer to the city center. Postcodes show that all the diarists we mention in this section live relatively close to the heart of the city. In Nick's discussion of entertainment options that are no longer available, he begins with a general discussion of cafes, explaining that he suffers from anxiety and felt ambivalent about cafes even before the pandemic. So when he moves onto missing pubs (10), he talks about what they represent (“such a cozy atmosphere”), rather than giving a faithful account of changes to his own routine. This idealization of the pre-lockdown possibility of going to a pub is then linked with the city (“such an Edinburgh experience”).

(10) Nick
“I still haven't really been to pubs. I mean, I know they're closed now, but I haven't really been to pubs, erm, very much because as we can see they're, they're not safe intrinsically, inherently. So those aspects, I mean ((it could)), coming into winter in Edinburgh it's actually very sad that the pubs closed, because, er, they represent such a cozy atmosphere, such a Edinburgh experience ((is)), you know, going to a Victorian wood-cladded pub with a fireplace perhaps and having a nice bi- bitter.”

Nick's commentary on pubs and what they represent is delivered in the present tense, compared to his narrative of lockdown, which is in the past tense (“nothing fundamentally changed”) with an occasional note in the historical present (“you're stuck. That's it. You stay inside”). As they reflect on their lives and routines before the government-imposed lockdown, in which traveling around the city was a regular feature of their day-to-day lives, these participants, all of them male, discursively construct Edinburgh as a place full of possibilities. The exercise of choice between these possibilities is an important part of their identity. The connection to gender is supported by corpus analysis^7^ which shows that in the entire set of diaries there is a quantitative tendency for men to use the word “choice” more than women, and other research (e.g., Collignon et al., 2021) showing less support for lockdown measures in the UK among men than among women. In their LDP diaries, these men look back to their quotidian activities pre-lockdown and long for the autonomy of that life. Fergus observes that their world has literally become smaller, but with the loss of choice it is metaphorically smaller too (“because your world becomes a lot smaller your choices diminish”) (11).

(11) Fergus
“Before the Lockdown, life was what now seems very different but then was normal. Life was very busy and it was all about freedom and more than anything choice … Erm, I work shifts so some days I might reward myself with a long lie, some days I may be up for work really early. I might go to the gym, erm, shopping I need to get in. Visiting people, maybe go outdoors for a walk. Life is very mixed. And, again, it's that freedom and that choice that I speak about. You could create your own day and almost overnight that that freedom was gone you could still- could still go outside during lockdown but the choice was gone. You no longer had the choice, “will I go to the gym? will I stay inside? will I go and see a friend? will I take a walk myself? will I go and visit sister and brother-in-law? will I go and visit parents?” that— the choice element, I think is the main difference, you know, your world becomes a lot smaller and because your world becomes a lot smaller your choices diminish. You–you can't choose to do the same things that you would normally do when you're only talking about being able to go into a much—a much smaller area than you would previously have at your disposal.”

Fergus clearly has difficulty situating the choices that he values about his lifestyle in the past. His first mention of pre-lockdown is in the past tense (“life was very busy”); but this is followed by the habitual (“I work shifts”). He lists the choices of pre-lockdown life in the subjunctive (“some days I might reward myself”) with occasional shifts to the present (“life is very mixed”); in some places he apparently abandons tense altogether (“visiting people”). With reference to the period of lockdown, he quotes his no-longer-available options in the future tense (“will I go to the gym?”).

In shifting to an impersonal “you” halfway through, he “tries to generalize his situation to that of others” (Piazza, 2019b). The locations given in (11) are rather generic (gym and shops), but later in the diary he changes the scale (Pritzker and Perrino, 2021), going from the loss of personal choices to the loss of choices “people” make in Edinburgh the city, panning out to the region, the country (12).

(12) Fergus
“Are people not gonna want to travel as widely around the city, around the region, around the country? are people not gonna want to meet up with friends, and maybe just go to an art gallery, go shopping, go for lunch because they're so accustomed to staying at home? Will we see an end to the Edinburgh Festival?”

The loss of the potential choices offered by the city leads Fergus to imagine the transformation of the city itself, and cities in general. The loss that he describes in (12), now experienced by a collective “we”, culminates in the imagined loss of the Edinburgh Festival, an annual, world-renowned arts and culture festival. The relationship of Edinburgh residents to the Edinburgh Festival is certainly complex, but the identity of the city is nevertheless bound up with the festival (Jamieson, 2004). The picture Fergus paints in (12) is of Edinburgh as a place in a dangerous flux – one whose identity is undergoing significant change and whose future is uncertain. The COVID-19 lockdown measures are ideologized as potentially dangerous, by extension.

## Discussion

Our analysis identified three ways in which those living-alone experienced a change in the organization of time and space before and during the first Covid-19 lockdown. In the first, international students are seen to shift away from their international student identity, with its highly structured time-space chronotope, taking on the outlook of a local, entitled to walk unspecified streets at their leisure. In the section Physical place can be replaced by virtual space, a small sample of retirees are seen to embrace a shift to virtual space in order to keep up their busy pre-lockdown social schedules. In the section Edinburgh was all about freedom and choice, a small sample of men living close to the city center experienced a loss of free unstructured time and space. While a robust analysis of demographic differences would require a larger sample size, the cases here demonstrate just some of the striking diversity in experiences of time and space in the first COVID-19 lockdown among residents of one geographic location.

We have talked about a shift in chronotopic orientation, rather than a metamorphosis or total transformation, because these subjects have not arrived at an entirely new identity: they are experiencing liminality, “the existential state of being caught between different times and spaces” (Piazza, 2019a, p. 3). Cox and Perry (2011) have described how, in the wake of disasters, and in the liminal period before a new identity is reconstructed, subjects struggle to put together identity markers. The COVID-19 lockdowns placed every member of society into a kind of liminality typically only experienced by the marginalized. Everyone was momentarily made to “strip[] off their ordinary identities, roles, and positions” (Eksner and Orellana, 2005, p. 2), and thrust into “the change process”, when a person is “in between two identity constructions: when they are neither one thing nor the other” (Beech, 2011, p. 286). The “world of the telling” (Perrino, 2005) in each LDP diary is a liminal space-time in which the individual reflects on both life-before-lockdown and life-in-lockdown. This “narrative practice” (De Fina, 2021) is also a “liminal practice” (Beech, 2011) where diarists negotiate new place identities. The relationship between structure and agency was radically impacted by the COVID-19 lockdowns, and individuals negotiate the resulting liminality through chronotopic discourse. While previous work has focused on liminality resulting from migration and displacement (e.g., Koven, 2019; Piazza, 2019b), here we see individuals responding to an unsettling liminality experienced within the home; forced sedentarism as opposed to forced mobility (see Britain, 2016).

As liminal practices, discourses around lifestyle changes express ideological positions on speakers' situation in relation to the pandemic. Their act of speaking is situated in material conditions (Park, 2021, p. 48, 50). The diarists of these three sections, though in a number of ways materially secure, are all in their own ways members of marginalized communities, whether due to race, nationality, age, or their shared characteristic of living-alone, excluded from many popular discourses of the pandemic, e.g., homeschooling, getting along with people in a confined space. Most of them go out of their way to signal that they feel fortunate in comparison to others who are suffering more for reasons related to the pandemic. Most also appear to embrace their liminality, as in (4) (“lovely…I really felt part of this place”) and (5) (“I really enjoy walking alone on a street now”). Or, they deny it altogether, as in (7) (“an excellent substitute for attending the service in the church and a fine opportunity to see new friends”) and (8).

At the same time, each speakers' diary simultaneously constructs a position on their marginalized status. For example, while the retirees in the section Physical place can be replaced by virtual space were able to maintain their pre-lockdown schedules by virtue of their access to resources and technology, their advanced age also made them especially susceptible to the worst effects of COVID-19. We suggest that narratives describing successful transitions to the virtual are not trivial, but rather that they enact a coping mechanism in a more existential sense: keeping busy will keep them inside and therefore will keep them alive. The overt figure of personhood here is the busy retiree, but the implied one is the stoic survivor.

In the section Edinburgh was all about freedom and choice we see liminality expressed through the construction of a place as a target of desire (Koven, 2019). The parallelism of Andrew's use of “I miss” (9) enacts the speaker's desires with an emphasis. The objects of those desires are all highly specific chronotopes, which together construct a before-lockdown storyworld. Directional expressions, “going to” and “trips to”, position Andrew as outside his desired chronotope (Koven, 2019). This is even more prominent in Fergus' narrative (11), where the desire to “go” and the inability to fulfill that desire is precisely what constructs a world that is “a lot smaller,” a description he uses three times in a parallel structure (Pritzker and Perrino, 2021, p. 380). Fergus is trapped between a nostalgia for the life that used to be, and to which he may not be able to return, and an uncertainty about what life will be like in the future. This is a typical expression of liminality: “home” is a place in the past or the future (Den Boer, 2015, p. 488); or home itself is “a longing for a nostalgic past or utopian future” (Al-Ali and Koser, 2002, p. 7).

In contrast, for Rajesh (6), the lockdown storyworld is constructed as “nothing,” the total lack of the implied “everything” that then characterizes the pre-lockdown storyworld. He stands out as orienting to a very different lockdown chronotope than the other international students, clearly suffering from the enforced liminality of the lockdown.

Interestingly, Nick (10) does not use any linguistic indicators of desire. While all three men quoted in the section Edinburgh was all about freedom and choice lament the loss of access to pubs, their narratives suggest contrasting ideologies toward the COVID-19 lockdown measures. Nick expresses an affective evaluation (“it's actually very sad”) about the restriction, but precedes this with a statement that aligns him with government health measures (“they're not safe”) and a claim to epistemological truth (“intrinsically, inherently”). In contrast, although Fergus makes no overt statements about emotion, his repetition of the world being “small” is clearly marked with negative affect, and is preceded by other statements of negative evaluation, e.g., that “freedom was gone” and “your choices diminish.” Based on this, we argue that Fergus' narrative expresses an anti-lockdown ideology and Nick's a pro-lockdown ideology. In liminal spaces, new identities are available for construction and negotiation. Other work has explored how these new identities connect to existing ones, specifically in terms of support for or against the UK COVID-19 lockdowns (e.g., Collignon et al., 2021).

For the international students, Edinburgh was a liminal place even before the lockdown: a temporary place of residence associated primarily with “the university” as place, time, and events. While the diary narratives in (4) and (5) associate walking the streets with ownership (“this place is also mine”), this simultaneously constructs their pre-lockdown experience as one of lack of ownership, drawing attention to their actual, highly temporary, status. Perhaps it is not so surprising, then, that their chronotopes suggest an ideology in favor of the lockdown policy; even Catherine's negative framing of her “very tiny room” (4) is immediately self-negated by a positive evaluation. Those social groups who experience limits on their agency in non-lockdown times may be more likely than others to produce lockdown chronotopes of opportunity, possibility, and belonging. The feeling of community becomes more available to them, even as it becomes less available to the dominant population.

Despite the small sample size, the contrast between most of the living-alone international students, on the one hand, and the Scottish men, on the other, is striking. Noting that all but one of the students who constructed a chronotope of possibility were female, a possible intersectional analysis emerges. The COVID-19 lockdown measures clearly restricted individual agency with respect to place. Men, in general, and especially white Scottish men, can be viewed as less used to restrictions on their place-based agency than women, especially immigrant women. In our dataset, Scottish men are more likely than immigrant women to comment on their loss of agency directly, and perhaps this is because it is quite literally more remarkable.

On a different note, it is striking that two students from Hong Kong [Patricia (5) and Siu Ming, not quoted here] chose to submit their diaries in Cantonese. Given that the Lothian Diary Project was associated with their university, we might expect them to deliver a diary in the English mode in which they are assessed. We suggest that the disruption of an international student identity and the liminality of lockdown means that their sociolinguistic identity can be more fluid, and they are able to use Cantonese as an index of non-student (or perhaps “real me”, Sharma, 2018) identity, even while describing a feeling of belonging in a non-Cantonese-speaking place.

The use of Cantonese is interesting in light of other material conditions at the time the diaries were recorded. In early 2020, rates of COVID-19 cases and movement restrictions in China were both frequent topics of discussion in European media. This heightened focus on China alongside a discursive framing of the COVID-19 virus as “Chinese” contributed to a spike in sinophobia and racist discourse and attacks targeting residents perceived to be Chinese: globally, nationally, and specifically in Edinburgh^8^, ^9^. One of the other Chinese participants, who was not part of the living-alone sample analyzed here, commented explicitly on feeling threatened if they walked outside. Although Chinese participants in the living-alone sample construct the lockdown chronotope explicitly in terms of the freedom to walk, and their sense of ownership, the material conditions of their act of speaking (Park, 2021, p. 48, 50) include real threats to Chinese students in Edinburgh that were taking place at the time. Furthermore, at the time of speaking, they are genuinely caught between two worlds: not safe in Edinburgh, not able to travel to China, and potentially not safe in China even if they did so. Chronotopes of belonging are therefore particularly striking among these speakers.

## Conclusion

In analyzing 36 audio/video diaries of Edinburgh and Lothian residents who lived alone during Scotland's first COVID-19 lockdown, we identified three sets of chronotopic orientation that seem characteristic of three demographic groups. International students experienced a loss of ‘the student experience' but were split between a lockdown experience that opened up the city to them and one that afforded no benefits. Retirees experienced lockdown through the transition from real to virtual space, emphasizing the similarities between the two and the ability to maintain pre-lockdown schedules. Men living near the city center experienced lockdown as a loss of freedom and choice, and their expression of this in some cases may be a reflection of their differing ideological positions toward government health measures.

These three groups represent 15 of the 36 diarists in the subsample; other demographic groups within the remaining speakers (e.g., gay/queer; disabled) did not show any consistent patterns of changes in chronotopic orientation. We will in future return to those speakers, as well as those who were not living alone, whose contributions go beyond the scope of the present paper.

The COVID-19 lockdown measures had a dramatic impact on personal experiences of space and place, across the world. By focusing on a diverse sample of individuals living alone in the Edinburgh area, we have shown how the first UK lockdown shifted experiences of belonging, and therefore, narratives of place and even ideologies of the pandemic response. These government-imposed restrictions on the occupation of space and places was not universally experienced as a loss of agency. Rather, pre-lockdown social differences, and the material conditions of those differences, resulted in dramatically contrasting chronotopes of lockdown life.

## Data availability statement

The datasets presented in this article are not readily available because Diaries are archived at the University of Edinburgh datastore. Diarists could choose to make their diaries public. These are shown on our website and at exhibits. Requests to access the datasets should be directed to claire.cowie@ed.ac.uk.

## Ethics statement

The studies involving human participants were reviewed and approved by PPLS Ethics committee, School of Philosophy, Psychology, and Language Sciences, University of Edinburgh. Written informed consent to participate in this study was provided by the participants' legal guardian/next of kin. Written informed consent was obtained from the individual(s) for the publication of any potentially identifiable images or data included in this article.

## Author contributions

ZE, NM, and SM collected the data. NM and AK processed the data. All authors contributed to the data analysis, which was led by CC. CC and LH-L wrote the paper, with feedback and contributions from the other authors. The analysis was conceived of and designed collaboratively among all authors. All authors contributed to the article and approved the submitted version.

## Funding

The following were sources of financial support: the ESRC Festival of Social Sciences; the Being Human Festival; the University of Edinburgh's College of Arts, Humanities, and Social Sciences Research Office; an ESRC Impact Acceleration Grant awarded to the University of Edinburgh; the School of Philosophy, Psychology, and Language Sciences' Knowledge Exchange and Impact Office; and the Center for Doctoral Training in Natural Language Processing, funded by the UKRI (EP/S022481/1) and the School of Informatics and School of Philosophy, Psychology & Language Sciences of the University of Edinburgh.

## Conflict of interest

The authors declare that the research was conducted in the absence of any commercial or financial relationships that could be construed as a potential conflict of interest.

## Publisher's note

All claims expressed in this article are solely those of the authors and do not necessarily represent those of their affiliated organizations, or those of the publisher, the editors and the reviewers. Any product that may be evaluated in this article, or claim that may be made by its manufacturer, is not guaranteed or endorsed by the publisher.

## Footnotes

^1^ https://www.ltg.ed.ac.uk/software/geoparser/

^2^With thanks to our colleagues on the Lothian Diary Project Team: Clare Llewellyn (School of Social and Political Science and Edinburgh Futures Institute) and Beatrice Alex (School of Literatures, Linguistics and English Language, and Edinburgh Futures Institute).

^3^ https://www.bbc.co.uk/news/uk-scotland-54161706

^4^Diarists who opted to be public are referred to by their first name, and others are provided with pseudonyms.

^5^https://www.bbc.com/worklife/article/20201023-coronavirus-how-will-the-pandemic-change-the-way-we-work, https://post.parliament.uk/the-impact-of-remote-and-flexible-working-arrangements/, https://www.theguardian.com/money/2021/dec/30/how-the-pandemic-transformed-the-world-of-work-in-2021.

^6^A “bubble” was “a network that links 2 households” (GOV UK) which was introduced during COVID-19 to allow for social support while also limiting social contagion. https://www.gov.uk/guidance/making-a-support-bubble-with-another-household.

^7^With thanks to our colleague Clare Llewellyn (School of Social and Political Science and Edinburgh Futures Institute).

^8^Example incident, 15 December 2020: https://www.edinburghnews.scotsman.com/news/crime/despicable-racist-attack-against-22-year-old-student-outside-university-edinburgh-library-3066434.

^9^Example incident, 15 March 2021: https://www.bbc.co.uk/news/uk-scotland-edinburgh-east-fife-5611304.
